# CUB domains are not required for OVCH2 function in sperm maturation in the mouse epididymis.

**DOI:** 10.1111/andr.13508

**Published:** 2023-08-08

**Authors:** Katarzyna Kent, Kaori Nozawa, Courtney Sutton, Frey Daniel, Masahito Ikawa, Thomas X. Garcia, Martin M. Matzuk

**Affiliations:** 1 Center for Drug Discovery, Baylor College of Medicine, Houston, TX 77030, USA; 2 Department of Pathology & Immunology, Baylor College of Medicine, Houston, TX 77030; 3 Department of Molecular and Human Genetics, Baylor College of Medicine, Houston, TX 77030, USA; 4 Department of Experimental Genome Research, Research Institute for Microbial Diseases, Osaka University, Suita, Osaka, Japan.; 5 Scott Department of Urology, Baylor College of Medicine, TX 77030, USA

**Keywords:** Sperm defects, male infertility, male contraceptive

## Abstract

**Background:**

Ovochymase 2 (*Ovch2)* is an epididymis-specific gene that is required for male fertility. While a multitude of reproductive tract-specific genes required for male fertility have been identified, OVCH2 is thus far the first protein required for male fertility that contains Complement C1r/C1s, Uegf, Bmp1 (CUB) domains located in tandem in the C-terminus of the protein. Identifying the functional significance of this unique domain has implications in better understanding fertility and infertility and as a potential contraceptive target.

**Objective:**

The goals of these studies were to understand the influence and requirement of OVCH2 CUB domains in the localization and functional requirement of OVCH2 in sperm maturation and function.

**Materials and methods:**

To this end, we performed *in vivo* localization analysis of OVCH2 and reproductive phenotype analysis of mice containing C-terminal FLAG tag on OVCH2, with either the entire protein intact, or CUB2 or both CUB1 and CUB2 genetically ablated. All mice were generated through the CRISPR/Cas9 gene editing approach.

**Results:**

We found that OVCH2 is specifically expressed in the proximal caput epididymidis, and absence of CUB2 did not affect this localization pattern. Although absence of both CUB domains significantly reduced sperm motility and progressive motility, this effect was not manifested in a reduction in fertility over a 6-month period mating trial, which showed no significant differences between control and CUB deletant mice. Further, absence of one or both CUB domains did not affect reproductive organ structure or sperm morphology.

**Conclusions:**

Our studies demonstrate that the CUB domains are not required for fertility in male mice, at least under the normal animal housing conditions our mice were tested in, and suggest that the enzymatic activity of the OVCH2 protease, in the absence of its CUB domains, is sufficient for normal sperm processing in the epididymis. Although our findings do not preclude the possibility that OVCH2 CUB domains are required under a yet identified stress condition, our findings demonstrate that the most likely region for deleterious mutations in men with idiopathic infertility and the most vulnerable site for inhibition of OVCH2 protein function is in its protease domain, and not its CUB domains. Our findings have implications in the genetic screening of infertile men and the development a novel non-hormonal male contraceptive by honing in on the more critical region of a functionally required protein.

## INTRODUCTION

Infertility affects 2.5–3.0 million couples in the U.S. yearly, of which the male factor is causative in 40–60 percent of the cases. However, about 30 percent of male patients seen for primary infertility evaluation are found to have idiopathic impairment of sperm function ^1,2^. In these cases, genetic abnormalities are highly suspected, although still not well understood. Genetic testing and genetic counseling are becoming a standard of care for men who present to the clinic with fertility problems. However, men with infertility in which the genetic cause has not been identified will not benefit from these procedures. Identification and characterization of fertility required genes is essential for making clinical translation from gene discoveries. Additionally, despite many couples desiring the ability to conceive a child, the global population continues to grow at an unsustainable rate, and we still lack effective means to control it. To date, the significant disparity between contraceptive options available to women and men has generated worldwide efforts into the development of the “male pill” to decrease the burden of family planning on women and to provide men with more contraceptive autonomy ^1,3^. Thus, identification and characterization of genes required for male fertility serves the dual role of further advancing diagnostic tests and treatment for male infertility, and the development of novel non-hormonal male contraceptives. In this study we investigate the cellular and molecular mechanisms governing the process of sperm development and maturation, with the aim to expand the diagnostic tools and genetic screens for idiopathic sperm dysfunctions, and to identify novel non-hormonal male contraceptive targets by capitalizing on recently identified male reproductive tract-specific genes coding for potentially druggable proteins vulnerable to inhibition by a small molecule.

In mammals, spermatozoa undergo a series of morphological, physiological, and biochemical modifications, during the process of sperm development (spermatogenesis and spermiogenesis) in the seminiferous tubules of the testes, and later during the process of sperm maturation in the epididymal lumen ^4–7^. Sperm maturation involves post-translational modifications, proteolytic processing (cleavage and/or activation), and positioning of key sperm membrane proteins, such as ADAM3, PRSS37, PRSS55, TMPRSS12, LYPD4, GALNTL5, PMIS2, CMTM2A, CMTM2B, to name a few ^8–14^. These maturational changes commensurate with sperm function and fertilizing ability in the female reproductive tract. Fully mature, fertilization-competent sperm is capable of transit through the harsh environment of the female reproductive tract, recognition, binding to, penetration, and fertilization of the oocyte. Sperm maturation is chiefly governed by epididymal factors in the luminal fluid which consists of a wide array of molecules, whose secretion, activity, and regulation are regionally distinct throughout the segments of the epididymis ^5,7,15^. Although the components or the proteome of epididymal duct fluid have been greatly explored and unique molecules have been identified ^4,15–19^, the epididymal factors involved in sperm maturation include currently uncharacterized proteins.

We identified Ovochymase 2 (*Ovch2*) as an epididymis-specific gene that is required for male fertility in mice and is likely involved in the process of sperm maturation ^20^. A global knockout (KO) of the *Ovch2* gene in mice results in complete male sterility in otherwise healthy mice, due to impairment of sperm transit through the uterotubal junction (UTJ) and failure to bind to the zona pellucida ^20^. Moreover, our *Ovch2* KO model demonstrated improper processing of sperm surface protein ADAM3, a disintegrin and metalloproteinase 3, suggesting potential protein-protein interaction (PPI) between OVCH2 and ADAM3 in the epididymis ^20^. Although we have placed OVCH2 into a novel NELL2-mediated lumicrine signaling pathway, with ROS1-ERK upstream regulation of OVCH2 secretion ^20^, the downstream interacting partners, or substrates of OVCH2 remain uncharacterized. *Ovch2* codes for a secreted trypsin-like serine protease and belongs to a large superfamily of proteins containing evolutionarily conserved C-terminally located Complement C1r/C1s, Uegf, Bmp1 (CUB) domains. The CUB domain is a structural motif of approximately 110 residues found almost exclusively in extracellular and plasma membrane-associated proteins, including proteases, many of which are developmentally regulated ^21–23^. In many non-reproductive tract specific proteins CUB domains are required for modulating proteolytic activity, recognition of specific substrates, and mediation of PPIs ^24–26^. Thus far, the significance of the CUB domain in male fertility has not been explored. The high degree of evolutionary conservation of CUB domains and close sequence similarities between mice and humans, suggests that CUB domains are functionally required in the extracellular OVCH2 protein. Although never demonstrated before to have any functional relevance in the male reproductive system, CUB domains have been implicated in rendering high substrate specificity, mediating protein-protein interactions, and modulating proteolytic activity in non-fertility related proteases ^24–26^. In a variety of extracellular proteins, CUB domains often occur in multiple copies and have been shown to associate with many different types of other domains ^21^. C-terminally located CUB domains are important both for substrate recognition and for regulating proteolytic activity ^24–26^, and appear to be involved in oligomerization and recognition of substrates and binding partners through a highly conserved calcium binding motif, Tyr-Glu-Asp-Asp, within the CUB domain ^27^. Proteins containing calcium-binding CUBs (cbCUB) have highly specific roles, and it is likely that these domains largely contribute to this specialization by conferring on them the ability to specifically recognize their protein ligands ^21,28^. CbCUB-mediated protein-ligand interactions usually involve multipoint attachment through several CUBs, resulting in high-affinity binding despite the low affinity of individual interactions ^21,29^. The functional significance of these low-affinity interactions was demonstrated by the fact that a single missense mutation of the conserved cbCUB region resulted in a severe pathological deficiency ^30^. CbCUB-mediated electrostatic interactions of CUBs are known to be strongly sensitive to low pH levels, raising the possibility of a decreased affinity in an acidic environment ^31^. This suggests a possible regulatory mechanism of fertility-related cbCUB proteins within the male and female reproductive tracts where the pH is graduated within different compartments ^32,33^. For instance, it was shown that the CUB domains of spermadhesins (SAs), seminal plasma proteins in cow and pig, bind carbohydrates on the oocyte zona pellucida when exposed to the alkaline environment of the female reproductive tract ^34–37^. Homologous, but inactive, copies of the boar spermadhesin genes are present in the human genome, while the corresponding region was lost from the mouse and rat genomes ^38–40^. OVCH2 belongs to a CUB domain-containing protein family with Tyr-Glu-Asp-Asp signature motif, indicating presence of a calcium-binding sites ^21^. The CUB domains of mouse and human OVCH2 may play evolutionary replacement roles to boar and pig SAs.

To dissect the molecular mechanisms of action of OVCH2, we examined herein whether OVCH2-CUB domains play a critical role in male fertility through mediating proteolytic activity of OVCH2 serine protease through recognition and binding to OVCH2 substrates during the process of sperm maturation in the epididymis. We designed a structure-function study in mice utilizing CRISPR/Cas9 gene editing strategy allowing for simultaneous epitope tagging, FLAG knock-in (KI), and deletion of significant portions of the OVCH2 protein (CUB2 in one mouse model, and both CUB domains in a separate mouse model). Data generated from these KI/KO mouse models have the potential of placing OVCH2 into a defined molecular, biochemical, and proteomic pathway that governs sperm maturation in the epididymis. These structure-function studies investigate whether the CUB domains are required or are relevant to OVCH2 interactions with sperm surface proteins, such as ADAM3, with the potential to identify a novel mechanism for the regulation of sperm maturation, highlighting vulnerability to inhibition within the region of the CUB or protease domains.

## MATERIALS AND METHODS

### Ethics statement

Mice were maintained in accordance with NIH guidelines, and all animal procedures were approved by the Institutional Animal Care and Use Committee (IACUC) at Baylor College of Medicine.

### Animals

For our in-house CRISPR/Cas9 gene editing procedures, B6D2F1 (BDF1) mice to harvest oocytes and sperm were purchased from Charles River (MA, USA), and timed-pregnant and pseudo-pregnant CD1 females and vasectomized CD1 males were obtained from Baylor College of Medicine’s Center for Comparative Medicine. Mice from our own in-house maintained BDF1 × BDF1 colony were used to mate with *Ovch2 FLAG* KI (*Ovch2*^*FLAG*^), *Ovch2 FLAG* KI and *CUB2* KO (*Ovch2*^*Δ2FLAG*^), and *Ovch2 CUB1* and *CUB2* KO (*Ovch2*^*Δ1,2*^) founder (F0) mice to expand each respective line. For phenotype analyses, sexually mature male mice were used. All mice were housed with a 12-h light:12-h dark cycle. All mouse experiments were performed according to the guidelines from the IACUC at Baylor College of Medicine. Our original *Ovch2* KO mouse line described in Kiyozumi et al. 2020^20^ was transferred from Osaka University to Baylor College of Medicine for further study.

### Human and mouse OVCH2 sequence alignment

Human OVCH2 (UniProt ID: Q7RTZ1) and mouse OVCH2 (UniProt ID: Q7M761) protein sequences were annotated and aligned using Needleman-Wunsch global alignment algorithm in SnapGene software version 6.1.1 (HEAD-26605).

### Generation and validation of *Ovch2* FLAG-tagged CUB knockout mice

Single guide RNA (gRNA) target sequences for mouse *Ovch2* were designed using the CRISPRdirect suite (https://crispr.dbcls.jp/), single-stranded oligo donor template (ssODN) for homology-directed repair (HDR) with FLAG KI and/or CUB KO were designed and ordered using Integrated DNA Technologies (https://www.idtdna.com/pages) (Supplemental Table S1). The custom sgRNAs were ordered (Sigma) and assembled into a ribonucleoprotein (RNP) complex with Cas9 protein (Thermo Fisher Scientific) at 37°C for 10 min. The final CRISPR cargo contained the RNP complex and the ssODN. Ova were harvested from the ampullae of super-ovulated BDF1 females and fertilized *in vitro* with caudal sperm harvested from a BDF1 male. CRISPR cargos for FLAG KI and CUB deletions were electroporated into zygotes using an ECM^™^ 830 electroporation system (BTX, Holliston, MA). Embryos were cultured overnight to the two-cell stage before being transferred into the oviducts of pseudo pregnant ICR mice. Founder mutations in pups born were identified by polymerase chain reaction (PCR) and Sanger sequencing. Founder mice with a +30 bp FLAG insertion sequence, −7,084 bp for CUB2 deletion, and −8,758 for CUB1 and CUB2 deletion, were used to expand each colony. Mice were genotyped by PCR with specific primers for the wild type (WT), FLAG KI, CUB2 and CUB1 KO alleles (supplemental Table S1). *Ovch2* cDNA was generated from caput epididymides of 16-week-old homozygous *Ovch2*^*FLAG*^, *Ovch2*^*Δ2FLAG*^, and *Ovch2*^*Δ1,2*^ males according to the protocols mentioned in RNA extraction and quantitative real time PCR section of materials and methods. For the PCR reaction, *Ovch2* cDNA-specific primers were used and are listed in the supplemental Table S2.

### RNA extraction and quantitative real time PCR

RT-qPCR was performed using 290 ng of total RNA extracted from caput epididymides of 16-week-old WT *Ovch2*, homozygous *Ovch2*^*FLAG*^, *Ovch2*^*Δ2FLAG*^, and *Ovch2*^*Δ1,2*^ mice (n=3). RNA extraction was performed according to the Direct-zol RNA Miniprep Plus kit by Zymo Research (cat. R2070T). Reverse transcription was performed according to the SuperScript IV First-Strand cDNA Synthesis Reaction Kit by Invitrogen (cat. 18091050). The RT-qPCR reactions were performed using the ThermoFisher TaqMan Gene Expression Master Mix (cat. 4399002) and TaqMan Gene Expression Assay (20X) probes specific for mouse *Ovch2* protease domain and mouse eukaryotic translation initiation factor 3 (*Eif3l*) as a housekeeping gene. The TaqMan assays used for specific transcripts are listed in supplementary material Table S2. Relative quantitative fold change was determined using the ΔΔCt method. In all analyses, the expression value of each gene was normalized to the amount of the internal control gene Eif3l^41^ cDNA to calculate a relative amount of RNA in each sample. RT-qPCR was carried out with 3 biological replicates per group, and each biological replicate was carried out in duplicate per plate. The raw critical threshold values of technical replicates were averaged before mean, standard error, and statistical analysis was determined for the biological replicates.

### Protein extraction and immunoblot analysis

Testes and epididymides from adult male mice were homogenized using VWR Bead Mill Homogenizer in Pierce IP buffer containing protease inhibitor followed by incubation for 1 hour at 4°C with gentle agitation. Subsequently, the lysate was sonicated for 10 s with 30 s rest interval at 20% amplitude and repeated four times. Lysates were cleared by centrifugation at 14,000 × g for 3 min at 4°C, and supernatants were subjected to sodium dodecyl sulfate-polyacrylamide gel electrophoresis (SDS-PAGE) under reducing conditions followed by blocking with Bullet Blocking One (Nacalai USA). The samples were then evaluated by immunoblot analysis with antibodies against FLAG (Proteintech, USA, 12940–1-AP) and OVCH2 (Sigma-Aldrich, ABS2248), GAPDH (Proteintech, HRP_60004) was used as a loading control. The chemiluminescent signal was developed using a ChemiDoc Imaging System (BioRad, USA).

### Immunoprecipitation analysis

Testes and epididymides protein lysates were generated according to the protein extraction protocol mentioned above, followed by 4 °C overnight incubation with magnetic beads coated with anti-FLAG antibodies (Invitrogen, Anti-DYKDDDDK Magnetic Agarose, A36797). The samples were then evaluated by immunoblot analysis with antibodies against FLAG (Sigma-Aldrich, A8592), OVCH2 (Sigma-Aldrich, ABS2248), and ADAM3 (Santa Cruiz, sc-365288). GAPDH (Proteintech, HRP_60004) was used as a loading control. The chemiluminescent signal was developed using a ChemiDoc Imaging System (BioRad, USA).

### PNGase F glycan cleavage of mouse OVCH2

Caput epididymis tissue from 16-week-old WT *Ovch2*, homozygous *Ovch2*^*FLAG*^, *Ovch2 Ovch2*^*Δ2FLAG*^, and *Ovch2*^*Δ1,2*^ mice was lysed as described previously in protein extraction and immunoblot analysis section of materials and methods. Protein concentrations were measured using Pierce BCA Protein Assay Kit (ThermoFisher Scientific, cat. 23225), and samples containing 15 μg of protein were digested with PNGase F according to the PNGase F Glycan Cleavage Kit protocol (ThermoFisher Scientific, cat. A39245). Undigested tissue lysates were used as control. The molecular weights of digested and undigested glycoproteins were determined using SDS-PAGE and immunoblot with anti-FLAG (Sigma-Aldrich, A8592) and anti-OVCH2 antibodies (Sigma-Aldrich, ABS2248).

### Immunofluorescence

For FLAG and OVCH2 immunostaining, epididymis and testis tissues were fixed overnight in 4% paraformaldehyde at 4°C and incubated in 10%, 15%, and then 20% sucrose. OCT-embedded 8 μm cryosections were mounted on plus slides, air-dried, and incubated in blocking solution comprised of phosphate buffered saline (PBS) containing 0.1% Triton X-100 (PBTx) with 5% normal donkey serum (NDS) and 3% bovine serum albumin (BSA). Slides were then incubated with anti-FLAG (Invitrogen, 8H8L17) or anti-OVCH2 antibody (Sigma-Aldrich, ABS2248) in blocking solution overnight; washed with washing solution comprised of PBTx containing 1% NDS and 3% BSA; incubated with Alexa Fluor 488-conjugated goat anti-rabbit IgG (Invitrogen) secondary antibody in blocking solution for 1 hour at room temperature; washed three times; subjected to a wash containing 1 μg/ml DAPI; a final wash; then coverslipped with ProLong Glass Antifade Mountant (Invitrogen). Stained tissues were then imaged using a Zeiss LSM 780 Confocal Microscope in the Optical Imaging & Vital Microscopy Core at Baylor College of Medicine.

### Male fertility assessment

Sexually mature control and mutant male mice were housed with two C57BL6J/129SvEv female mice for 6 months. During the mating period, the number of pups born per litter per male was counted at 2-month and 6-month time point. The total number of litters and pups per male over the mating trial was calculated and divided by the number of months to generate averages and statistics per genotype. The average number of pups per litter is based on the average litter size per male where a litter is defined as one or more pups.

### Histological analysis of reproductive organs

Testes and epididymides from WT *Ovch2*, homozygous *Ovch2*^*FLAG*^, *Ovch2*^*Δ2FLAG*^, and *Ovch2*^*Δ1,2*^ mice were collected and fixed in Bouin’s fixative (Sigma-Aldrich) overnight at room temperature and washed in 70% ethanol to remove excess fixative. Tissues were processed on automated Sakura VIP 5 processor, embedded in paraffin, sectioned at 4-μm thickness, and stained by Periodic Acid-Schiff (PAS)-Hematoxylin. Entire slides were scanned at 40X with an Aperio AT2 slide scanner (Leica Microsystems). Epididymis longitudinal sections and testis cross-sections were examined for morphological changes, additionally, testis cross-sections were examined for stages of spermatogenesis in accordance with the standard staging criteria ^42,43^.

### Analysis of sperm parameters and sperm kinematics

Sperm were extracted by mincing a single cauda segment of epididymis 20 times in Enhance Sperm Wash w/Gentamicin (Vitrolife, Sweden) medium at 37°C. After a 15-minute incubation, an aliquot of supernatant was diluted and applied to a 20 μm-depth Leja semen analysis slide (Spectrum Technologies, Healdsburg, CA), and the sperm number, motility, progressive motility, hyperactivation and sperm kinematics parameters were measured using a Hamilton Thorne CEROS II system for computer assisted sperm analysis (CASA). Sperm parameters were measured again after a 90-minute incubation at 37°C, to allow for capacitation.

### Sperm viability analysis

WT *Ovch2*, homozygous *Ovch2*^*FLAG*^, *Ovch2*^*Δ2FLAG*^, and *Ovch2*^*Δ1,2*^ mouse sperm viability was assessed as previously described using the LIVE/DEAD^™^ Sperm Viability Kit by Thermo Fisher ^44^. Briefly, freshly released cauda epididymal sperm suspension was collected and diluted to 3 × 10^6^ cells/mL in 1.0 mL in Enhance Sperm Wash w/Gentamicin (Vitrolife, Sweden) medium in a 2-mL microcentrifuge tube at 37°C. SYBR-14 and propidium iodide dyes were included in the LIVE/DEAD sperm viability kit to stain live spermatozoa (green) and dead spermatozoa (red) as reported ^45,46^. To each sperm suspension, SYBR-14 was diluted to a 100-nM final concentration and incubated for 10 min at 37 °C. After the incubation period, propidium iodide was diluted to a 12-μM final concentration in each sperm suspension and incubated for 10 min at 37 °C. A 20-μL sample was pipetted onto a SuperFrost microscope slide for each sample, coverslipped, and imaged with an epifluorescence microscope. At least 100 cells were counted per mouse and 500 cells counted per genotype. Cells were manually counted with ImageJ software (National Institutes of Health) Cell Counter plugin.

### Statistical analysis

Statistical significance was evaluated using the two-tailed unpaired Student t test assuming unequal variances except as otherwise noted. Data are represented as means ± SEM. Asterisks represent the level of significance: *P < 0.05; **P < 0.01; ***P < 0.005; ****P < 0.0005; ns, not significant.

## RESULTS

### OVCH2 protein structure is highly conserved between mice and humans.

**Fig. 1.**
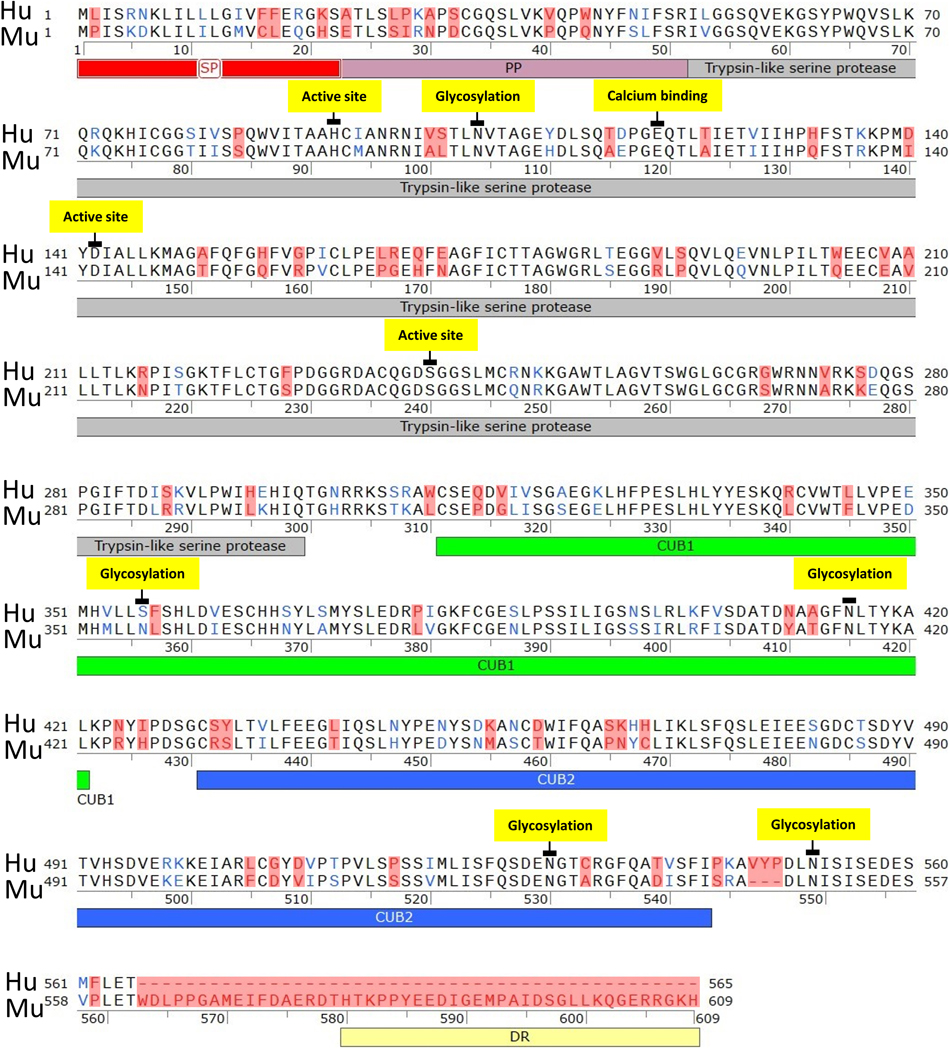
Human and mouse OVCH2 proteins are conserved. Human and mouse protein sequences were exported from and annotated according to data available in UniProt (Q7RTZ1, Q7M761, respectively). Pairwise protein sequence alignment was performed in SnapGene software (version 6.1.1) using Needleman-Wunsch global alignment algorithm. Structural domains of OVCH2 are color coded and labeled: SP= signal peptide, PP= propeptide, DR= disordered region. PTM sites are highlighted yellow and show 100% sequence identity. Amino acids colored blue belong to the same group with identical side chain polarity. Amino acids colored red denote dissimilarities between the human and mouse proteins.

For novel and mostly understudied genes, one can gain insight into probable protein function by analyzing sequence and protein structure similarity with other known proteins. Further, there is growing evidence showing that posttranslational modifications may in fact be equally important for determining and maintaining the function of a protein ^47^. Our sequence analysis of human and mouse OVCH2 reveal striking similarities in both coding regions and predicted post-translational modification sites (PTMs), suggesting a high degree of conservation and confidence in the functional prediction (Figure 1). Human and mouse gene orthologs share greater than 60% sequence identity and over 80% amino acid sequence homology in the protease domain, and a greater than 70% sequence homology between the tandem CUB domains. PTM sites in both human and mouse OVCH2 proteins are highly conserved (Figure 1) suggesting high confidence in functional similarity.

### Generation of *Ovch2* FLAG KI and *Ovch2* FLAG KI CUB KO mice

**Fig. 2.**
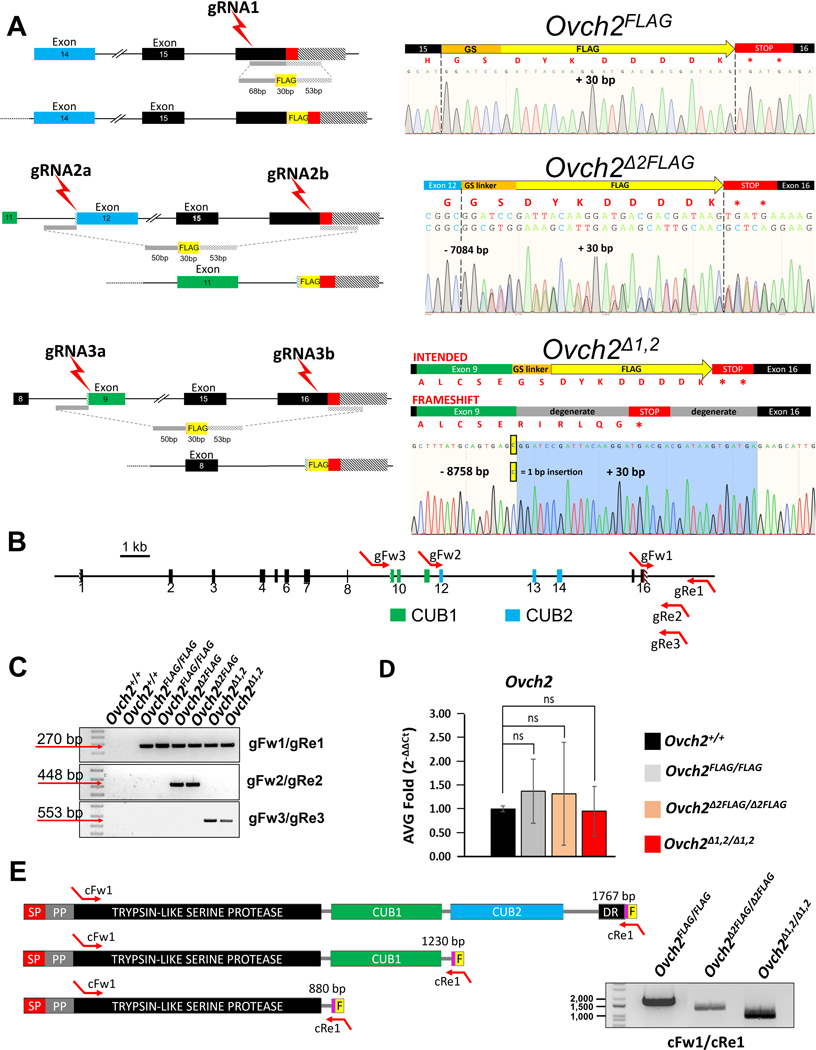
Generation and validation of the *Ovch2* FLAG KI, and CUB KO mice. (A) CRISPR/Cas9 gene editing strategy of generating *Ovch2* FLAG KI, and CUB KO mice, locations of the single guide RNA (gRNA) and Cas9 cleavage sites are indicated (red bolts), along with the homology arms for the single stranded oligo donor templates (ssODN) for homology directed repair (HDR) (left). Sanger sequencing chromatograms (right) confirm the predicted theoretical sequences of the founder (F0) generation of mutant mice. (B) Genomic structure of the mouse *Ovch2* locus to scale (1 kb) with aligned genotyping primers forward and reverse (gFw/gRe). CUB1 and CUB2 coding regions are marked green and blue, respectively. (C) Genotyping of *Ovch2* alleles. Primers shown in Figure 1B amplify specific amplicons for the FLAG KI (FLAG - 270 bp) or CUB KO alleles (Δ2FLAG - 448 bp, or Δ1,2 – 553 bp). The sizes of several DNA ladder bands are shown for comparison. (D) Caput epididymides RT-qPCR expression analysis of WT *Ovch2*, homozygous *Ovch2*^*FLAG*^, *Ovch2 Ovch2*^*Δ2FLAG*^, and *Ovch2*^*Δ1,2*^ mice (n=3) showing no significant (ns) differences in the average fold change across the control and experimental groups. *Eif3l* was used as expression control. (E) Diagrams of theoretical cDNA sequences of each mutant mouse with respective transcript lengths (bp), showing localization of the cDNA-specific primer set (cFw1 and cRe1). Agarose gel image with cDNA amplicons from caput epididymides of each of the mutant mice. The sizes of several DNA ladder bands are shown for comparison.

In our previous studies, we identified OVCH2 as a conserved epididymis-specific protease required for male fertility^20^ with knockout male mice phenocopying gene deletants of other sperm-specific proteases such as ADAM3, PRSS37^48^, PRSS55^10^, and TMPRSS12. Since OVCH2 is the only protease among these to contain CUB domains, we sought to evaluate the involvement of the CUB domains in OVCH2 function and processing of sperm surface proteins by deleting one and both CUB domains. Since the only available anti-OVCH2 antibody recognizes the portion of the protein removed when the CUB domains are deleted, we also inserted a FLAG (DYKDDDDK)-tag at the C-terminus of CUB deletant mice to detect CUB deletant proteins in mutant mouse tissues (Figure 2A). Since the FLAG-tag would also enable us to perform valuable *in vivo* pulldown experiments—experiments that we found the anti-OVCH2 antibody to be insufficient for—we also added the FLAG-tag sequence to wild-type, full-length OVCH2. Using our previously described CRISPR/Cas9 gene editing strategy, we included ssODNs in our electroporation of embryos to induce HDR to achieve sequence-specific repair after induction of double stranded breaks (DSBs).

As illustrated in Figure 2A, to generate the FLAG-tagged, full-length *Ovch2* allele (*Ovch2*^*FLAG*^), we designed and utilized one single guide RNA (gRNA), to generate a DSB near the C-terminus, and an ssODN, to insert the FLAG-tag through HDR. To generate the CUB2 deletant with a C-terminal FLAG-tag (*Ovch2*^*Δ2FLAG*^) and combined CUB1 and CUB2 deletant with C-terminal FLAG-tag (*Ovch2*^*Δ1,2FLAG*^), the design necessitated a second gRNA towards the start of each desired CUB deletion, either in exon 12 of *Ovch2*, for CUB2-only deletion, or in exon 9 of *Ovch2*, for combined CUB1 and CUB2 deletion (Figure 2A). gRNA and ssODN sequences for each of the mutants are provided in Supplementary Table 1. One-cell stage embryos were electroporated with Cas9 protein, gRNA, or gRNAs, and ssODN, and the one-cell stage embryos were then transferred into the oviducts of pseudo-pregnant females.

After Sanger sequencing-based screening of all founders obtained, we were able to acquire each of the desired mutant alleles described above, except *Ovch2*^*Δ1,2FLAG*^. We obtained a *Ovch2*^*FLAG*^ allele that contains an in-frame 30-bp GS-linker plus FLAG sequence prior to the STOP codon (Figure 2A), and an *Ovch2*^*Δ2FLAG*^ allele that contains a 7,084-bp deletion spanning the CUB2 sequence with an in-frame 30-bp GS-linker plus FLAG sequence prior to the STOP codon (Figure 2A). Although we were able to obtain a founder containing the desired 8,758-bp deletion that ablates both CUB1 and CUB2, this founder contains a confirmed single nucleotide insertion—just prior to the 30-bp GS-linker and FLAG sequence—that introduces an unintended frame shift and premature STOP codon within the FLAG coding sequence, ablating the FLAG tag (Figure 2A). Since this *Ovch2*^*Δ1,2*^ allele (without functional FLAG-tag; appropriated named without the FLAG designation) could still be used for studies assessing the requirement of both CUB domains, we proceeded with expanding and utilizing this line. Specific primers for WT and mutant alleles were designed as depicted in Figure 2B, and used for subsequent genotyping with representative results shown in Figure 2C. Through real-time PCR, with a TaqMan probe that binds universally within the protease domain-encoding sequence, we were able to show that each of the FLAG-tagged and/or CUB deletant alleles are expressed at mRNA levels not significantly different than wild-type *Ovch2* in caput epididymides (Figure 2D). In lieu of a functional FLAG-tag in *Ovch2*^*Δ1,2/Δ1,2*^ mice for immunodetection and confirmation that the protein is made, we used semi-quantitative PCR of reverse transcribed caput epididymides RNA, with primers amplifying the near full-length (~93%) of coding sequence of each mutant transcript (cDNA primer sequences are shown in supplemental Table S2), to confirm that all transcripts, including *Ovch2*^*Δ1,2*^, are appropriately made and at their predicted sizes (Figure 2E). As shown in Figure 2E, our amplification strategy obtained—from start codon to stop codon in each transcript—bands corresponding to 1,860 bp out of 1,957 bp for *Ovch2*^*FLAG*^, 1,323 bp out of 1,420 bp for *Ovch2*^*Δ2FLAG*^, and 973 bp out of 1,070 bp for *Ovch2*^*Δ1,2*^.

### Synchronous expression of OVCH2-FLAG fusion protein and endogenous OVCH2

**Fig. 3.**
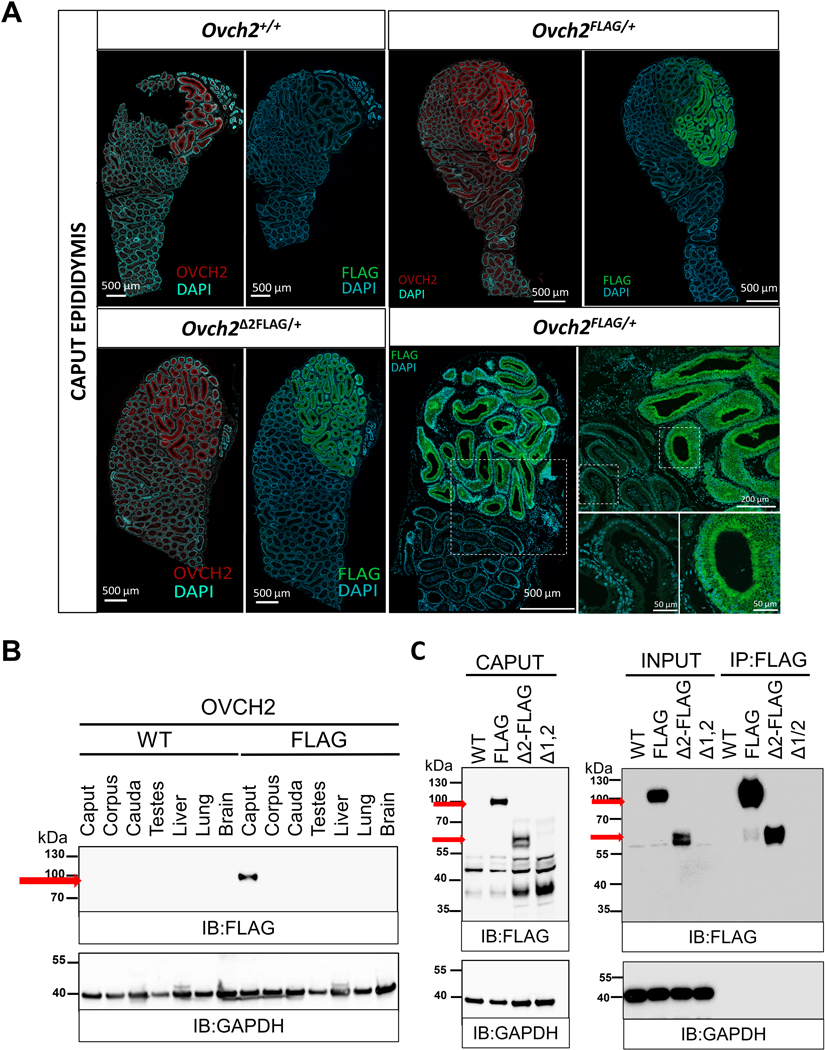
Synchronous expression of OVCH2-FLAG, OVCH2-CUB KO and endogenous OVCH2. (A) OVCH2-FLAG, OVCH2Δ2-FLAG fusion proteins and endogenous OVCH2 protein expression specificity in the caput epididymis of the KI/KO heterozygous mice at the age of 16 weeks (n=3). OVCH2 and FLAG immunostaining (red and green, respectively) of frozen sections showing overlapping localization specifically restricted to the luminal side of the proximal caput epididymis. Images of entire caput epididymides were taken with a Zeiss Axio Imager. M2 with Plan-Apochromat 10x/0.45 objective. Higher magnification inset images (lower right panels) were taken separately with a Zeiss LSM 880 Airyscan FAST Confocal Microscope with Plan-Apochromat 40x/1.4 Oil DIC M27 objective. Higher magnification images were rotated 90 degrees clockwise and flipped along the vertical axis relative to images of entire caput epididymides. DAPI (light blue) was used as the nuclear stain. Scale bars are shown for reference: 500 μm, 200 μm, and 50 μm. (B) Immunoblot with tissue panel of reproductive and non-reproductive tissue lysates from wild type (WT) and homozygous *Ovch2*^*FLAG*^ (FLAG) males showing restricted expression to the caput epididymis in OVCH2-FLAG fusion protein (at ~90 kDa). GAPDH was used as a loading control. (C) FLAG immunoblot (left) of caput epididymis lysates from WT *Ovch2*, *Ovch2*^*FLAG*^*, Ovch2*^*Δ2FLAG*^*, Ovch2*^*Δ1,2*^ homozygous mice. Immunoprecipitation with FLAG pull-down showing enrichment for OVCH2-FLAG fusion proteins (right). Predicted molecular weights of OVCH2 proteins is indicated by red arrows, ~90 kDa for OVCH2 FLAG, ~60kDa for OVCH2Δ2-FLAG. GAPDH was used as a loading control. WT= wild type; FLAG=FLAG KI; Δ2-FLAG= CUB2 KO, FLAG KI; Δ1,2= CUB1/2 KO.

In addition to semi-quantitative and real-time PCR to examine transcript expression, we used immunofluorescence microscopy and immunoblot analysis of WT (*Ovch2*^+/+^), heterozygous (*Ovch2*^*FLAG*/+^, and *Ovch2*^*Δ2FLAG*/+^), and homozygous (*Ovch2*^*FLAG/FLAG*^ and *Ovch2*^*Δ2FLAG/Δ2FLAG*^) FLAG-tagged mutant mice, to examine protein expression and localization in the epididymis. Through immunofluorescence analysis of testis and epididymis cryosections from heterozygous and homozygous mice, we found that OVCH2-FLAG protein stained by α-FLAG and α-OVCH2 antibodies, and OVCH2-Δ2FLAG protein, stained with α-FLAG antibody, are expressed by epithelial cells, presumably principal cells, of the proximal caput epididymidis, in a pattern that is indistinguishable between WT OVCH2 protein stained with α-OVCH2 antibody (Figure 3A). Absence of FLAG staining in *Ovch2*^+/+^ mice demonstrates specificity of α-FLAG antibody staining in *Ovch2*^*FLAG*/+^ and *Ovch2*^*Δ2FLAG*/+^ mice, while absence of OVCH2 staining in *Ovch2*^*Δ2FLAG/Δ2FLAG*^ As expected, neither α-FLAG nor α-OVCH2 antibodies resulted in a positive signal in *Ovch2*^*Δ1,2/Δ1,2*^ mice, which is consistent with the α-OVCH2 antibody epitope corresponding to the last 179 amino acids within the deleted CUB, and the premature stop codon within the FLAG coding sequence caused by a single nucleotide insertion described above and shown in Figure 2A. Thus, our immunofluorescence staining results demonstrate that neither inclusion of a C-terminal FLAG-tag, nor deletion of CUB2, with inclusion of a C-terminal FLAG-tag, affect protein expression or localization of OVCH2 in the epididymis. Through immunoblot analysis of various reproductive and non-reproductive tract tissues, and FLAG immunoprecipitation of caput tissue lysates, we further confirmed caput-specific expression of OVCH2-FLAG and OVCH2-Δ2FLAG proteins in the epididymis, with molecular weights of the detected proteins consistent with that of predicted molecular weights, when taking into account the addition of PTMs – glycosylation (Figure 3B and 3C). In our study we observed that the molecular weight (MW) of mouse OVCH2 protein deviates from the estimated value (65.2 kDa for WT, 66.3 kDa for FLAG KI, 46.3 kDa for Δ2FLAG, and 32.6 kDa for Δ1,2 OVCH2), which is evident in our anti-FLAG and anti-OVCH2 immunoblots (Figure 3, 6, S1). Both mouse and human OVCH2 are glycoproteins with predicted 4 to 5 N-glycosylation sites within the protease and CUB domains (Figure 1), and the MW of each glycan chain is predicted to be 3.6 to 4.5 kDa on average^49^. Previous studies demonstrated that the determination of MWs of glycosylated proteins is often hampered by the attached N-Glycan chains, whose individual MW and the number of N-glycosylation sites present in the protein sequence can impact protein migration during SDS-PAGE analysis^49^. Such deviations, which are largely due to the decrease in migration of glycoproteins resulting from poor glycan–SDS interactions, are frequently encountered in MW determination of most glycoproteins using SDS-PAGE^49^. Since most N-linked oligosaccharides can be released from proteins using PNGase F, we performed PNGase F digestion on caput tissue lysates from *Ovch2* WT and mutant mice to investigate the impact of N-glycosylation on the predicted MW of mouse OVCH2 protein and at the same time validate the intended mutations within *Ovch2* (Figure S1). We found that after the PNGase digestion the MW of WT and mutant OVCH2 decreased approximately 20kDa, which corresponds to the 4–5 predicted N-glycosylation sites within the protein sequence (Figure S1). Taken together, while we cannot prove through direct measurement that OVCH2-Δ1,2 protein is made in *Ovch2*^*Δ1,2/Δ1,2*^ mice, our results confirm expression of OVCH2-FLAG and OVCH2-Δ2FLAG proteins from *Ovch2*^*FLAG*/+^ and *Ovch2*^*Δ2FLAG*/+^mice, respectively, and further shows that these proteins—despite significant sequence alterations, especially for the CUB2 deletant missing a significant portion of its protein—continue to be expressed and mimic endogenous OVCH2 expression, suggesting that, at minimum, *Ovch2*^*FLAG/FLAG*^ and *Ovch2*^*Δ2FLAG/Δ2FLAG*^ mice may serve as useful genetic tools to investigate the physiological function and molecular mechanism of OVCH2, and the functional relevance of their CUB domains in male reproduction.

### *Ovch2* FLAG KI and *Ovch2* CUB KO mice exhibit normal fertility.

**Fig. 4:**
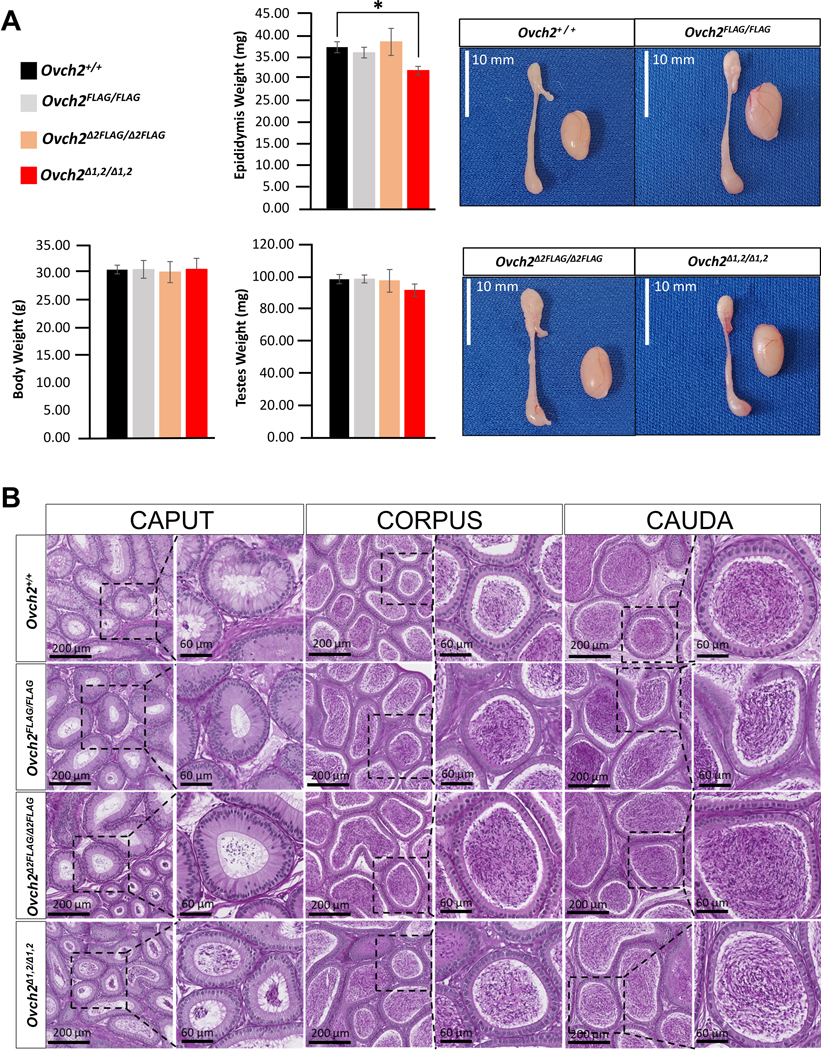
Mice lacking both OVCH2 CUB domains have smaller but structurally functional epididymis. (A) gross morphology of reproductive organs from 16-week-old homozygous *Ovch2*^*FLAG*^, *Ovch2*^*Δ2FLAG*^, *Ovch2*^*Δ1,2*^ males and *Ovch2* WT littermates (n=5). The average body and testes weight were similar across groups. The average epididymis weight from CUB1/2 deletion males (red bar) decreased significantly (p-value = 0.03). (B) Histological analysis of the epididymal segments (caput, corpus, cauda) from homozygous *Ovch2*^*FLAG*^, *Ovch2*^*Δ2FLAG*^, *Ovch2*^*Δ1,2*^ males and *Ovch2* WT littermates (n=3). Bouin’s fixed, 4μm-longitudinal sections of epididymis were stained with PAS-hematoxylin. Each square represents one of the epididymal segments (right) with a magnified view of selected areas (left). Scale bars are added for reference.

**Fig. 5.**
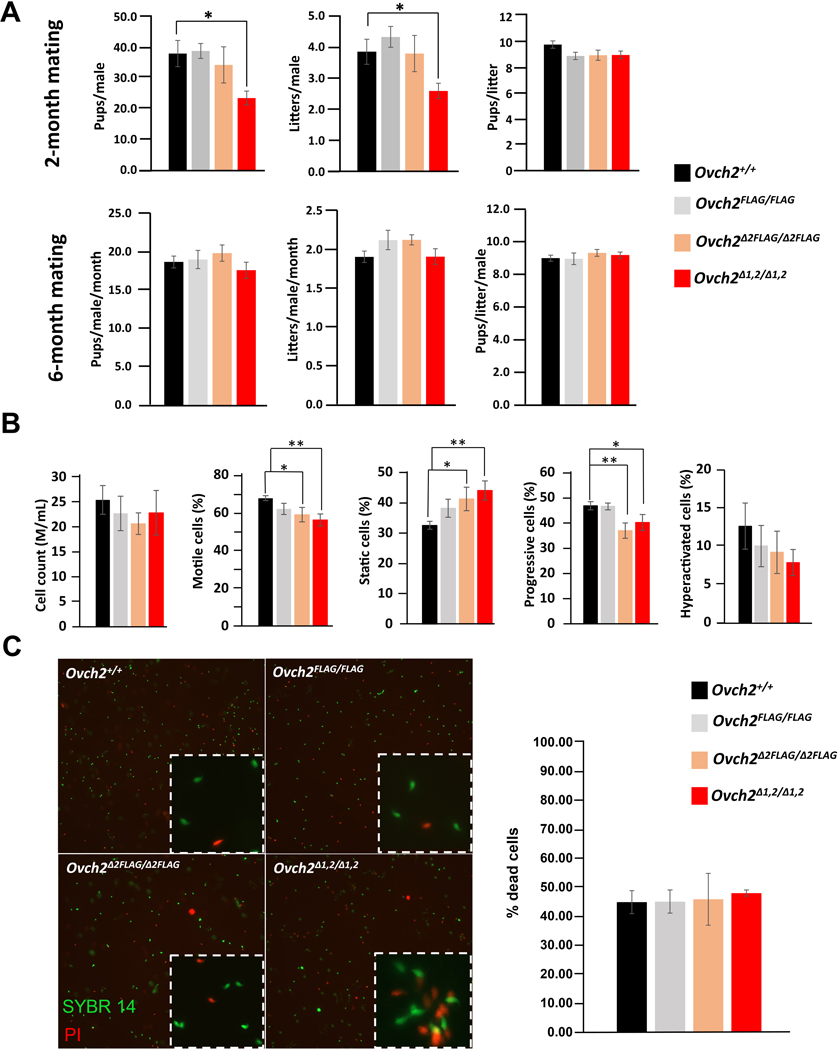
Mice lacking OVCH2 CUB domains exhibit decreased sperm motility but remain fertile. (A) average pup and litter count of homozygous *Ovch2*^*FLAG*^, *Ovch2*^*Δ2FLAG*^, *Ovch2*^*Δ1,2*^ males and *Ovch2* WT littermates (n=5). Initial decrease in fertility in CUB1/2 KO males (2-month mating) was restored to levels comparable to those in WT *Ovch2*, *Ovch2*^*FLAG*^ and *Ovch2*^*Δ2FLAG*^ males after 6 months of mating trials. (B) Computer assisted sperm analysis showing decrease in the average sperm motility and progressive motility in homozygous *Ovch2*^*Δ1,2*^ males. The average cell count and the percentage of hyperactivated cells were not significantly different across the experimental and control groups. (C) sperm viability assay showing normal levels of cell death across the experimental and control groups. Asterisks indicate significance levels: *P < 0.05; **P < 0.01; ***P < 0.005; ****P < 0.0005.

To begin to assess the effect of ablation of the CUB domains on male mouse fertility, we analyzed the weight, morphology, and histology of the testes and epididymides isolated from 16-week-old *Ovch2*^+/+^, *Ovch2*^*FLAG/FLAG*^, *Ovch2*^*Δ2FLAG/Δ2FLAG*^, and *Ovch2*^*Δ1,2*^ males. We found no significant difference in the average body weight or testes weight, whereas the average epididymis weight was significantly lower in the males missing both CUB domains (p-value = 0.04) (Figure 4A). Interestingly, upon histological analysis we did not detect any differences in the structure or composition of the tissue of the epididymal segments: caput, corpus, or cauda (Figure 4B). To determine whether our mutant mice experienced any spermatogenic defects, we performed a thorough histomorphometry analysis of the testis cross sections at all 12 stages of spermatogenesis. We found no significant differences between mutant and control males at any stages of spermatogenesis (Figure S2). To test fertility, beginning at 6 weeks of age, sexually mature *Ovch2*^+/+^, *Ovch2*^*FLAG/FLAG*^, *Ovch2*^*Δ2FLAG/Δ2FLAG*^, and *Ovch2*^*Δ1,2/Δ1,2*^ males (*N* ≥ 5 per genotype) were housed with two WT C57/129 females continuously each for a total of 6 months. At the 2-month time point of the mating trials control mating pairs had 38.1 pups on average, homozygous CUB2 deletion males had 34.4 pups, while homozygous CUB1/2 deletion males had 23.6 pups on average, which was significantly lower than their littermates (p-value = 0.02) (Figure 5A). However, after 6 months of test mating trials, we found that *Ovch2*^*FLAG/FLAG*^, *Ovch2*^*Δ2FLAG/Δ2FLAG*^, and *Ovch2*^*Δ1,2/Δ1,2*^ males sired a number and size of litters that was not significantly different from *Ovch2*^+/+^ controls (Figure 5A). Collectively, this data shows that ablating CUB2 and CUB1 and CUB2 does not affect the cellular architecture nor function of the reproductive organs and shows that the CUB domains of OVCH2 are not the critical component of OVCH2 that is required for male fertility.

### Sperm of mice lacking OVCH2 CUB domains remain functional despite having decreased motility and velocity.

To assess the effect of the CUB domain deletion on key sperm parameters, we performed computer-assisted sperm analysis (CASA) of sperm isolated from caudae epididymides of 16-week-old control and homozygous mutant males (*N*=5 each genotype). The sperm counts from caudae epididymides of *Ovch2*^*FLAG/FLAG*^, *Ovch2*^*Δ2FLAG/Δ2FLAG*^, and *Ovch2*^*Δ1,2/Δ1,2*^ males showed no significant differences in comparison to *Ovch2*^+/+^ males (Figure 5B). However, the percentage of motile sperm after a 15-minute incubation was significantly decreased in both *Ovch2*^*Δ2FLAG/Δ2FLAG*^, and *Ovch2*^*Δ1,2/Δ1,2*^ males (*P*=0.03 and 0.002, respectively) compared to both *Ovch2*^+/+^ and *Ovch2*^*FLAG/FLAG*^ males (Figure 5B, Vid. S1). The percentage of sperm exhibiting progressive motility after a 15-minute incubation was also significantly decreased in both *Ovch2 CUB2* and *CUB1/2* KO mice (*P*=0.001 and 0.04, respectively) compared to their control littermates (Figure 5B). We then analyzed the sperm kinematic parameters, average path velocity (VAP), curvilinear velocity (VCL), straight-line velocity (VSL), and found that after a 15-minute incubation the VAP and VSL were significantly decreased in *Ovch2*^*Δ2FLAG/Δ2FLAG*^ males (*P*=0.006 and 0.01, respectively) while after 90 minute incubation VAP, VCL and VSL remained significantly lower in *Ovch2*^*Δ2FLAG/Δ2FLAG*^ mice compared to their littermates (*P*=0.001, 0.01, and 0.009, respectively) (Figure S3). Based on these findings, we next sought to determine whether these decreased sperm parameters in some mice could be attributed to increased cell death rather than impaired motility. To address this, we performed sperm viability assay on 16-week-old control and homozygous mutant males (*N*=3 each genotype) and found that the average percentage of dead sperm stained with propidium iodine (red) did not significantly differ across the four groups of mice (Figure 5C). Taken together, the abnormal sperm parameters in the *Ovch2 CUB* deletion mice could not likely be attributed to the increases in sperm death. Nevertheless, the abnormal sperm remained functional, given that the mutant males sired offspring in numbers comparable to their control littermates.

### *Ovch2* CUB domains deletion does not impair ADAM3 processing in the mouse epididymis.

**Fig. 6.**
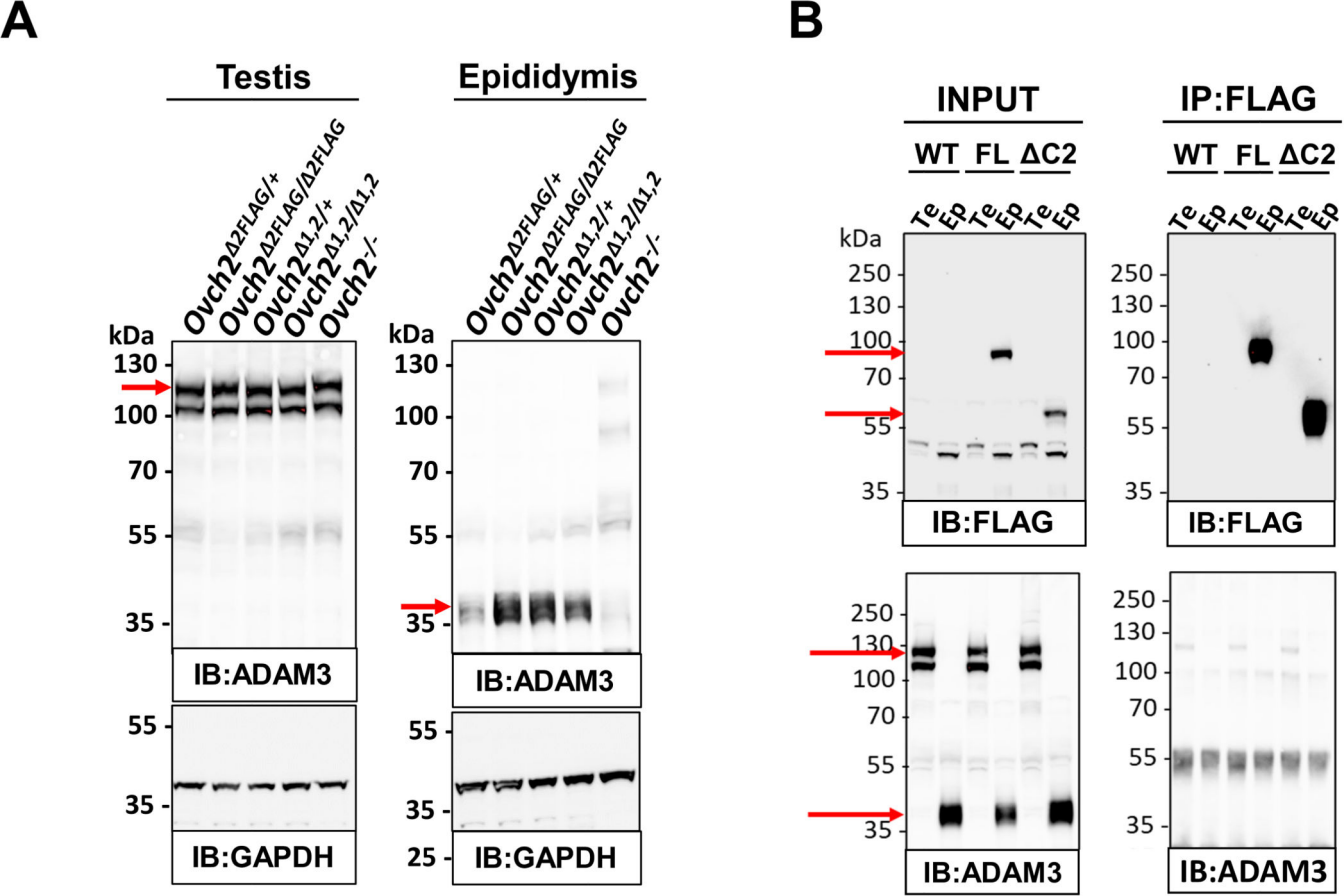
Processing of sperm surface protein ADAM3 in mice lacking OVCH2 CUB domains. (A) Immunoblot analysis of testis and epididymis tissue lysates from heterozygous and homozygous *Ovch2*^*FLAG*^, *Ovch2*^*Δ2FLAG*^, *Ovch2*^*Δ1,2*^ males showing ADAM3 prior to cleavage (~110 kDa) in the testis and cleaved (mature) ADAM3 (~42 kDa) in the epididymis. Tissue lysates from homozygous *Ovch2* KO mice (*Ovch2*^−/−^) were used as a control of aberrant ADAM3 processing. GAPDH was used as a loading control. (B) FLAG immunoprecipitation analysis of testes (Te) and epididymis (Ep) tissue lysates from wild type (WT), homozygous *Ovch2* FLAG KI (FL), and homozygous FLAG KI/CUB2 KO (ΔC2) mice. FLAG pulldown and FLAG immunoblot (top right) show predicted drop in molecular weight of FLAG-tagged full length OVCH2(~90 kDa) and FLAG-tagged OVCH2 without CUB2 domain (~60 kDa). ADAM3 immunoblot (bottom right) shows immature ADAM3 protein in the testes at ~110 kDa and cleaved ADAM3 in the epididymis at ~42 kDa (red arrows). No specific ADAM3 bands were detected in the FLAG pull-down (bottom right).

We postulated that the CUB domains likely modulate the proteolytic activity of the OVCH2 trypsin-like serine protease through recognition of and binding to OVCH2 substrates. Given that a global KO of the *Ovch2* gene resulted in abnormal processing of ADAM3 in mice, we investigated whether we could recapitulate this finding in our CUB deletant mice, thus showing involvement of the CUB domains in OVCH2-ADAM3 interaction. To detect the levels of OVCH2 and ADAM3 proteins, and to assess abnormalities in ADAM3 proteolytic cleavage in the epididymis, we isolated lysates from testes and epididymides tissues of 16-week-old *Ovch2*^+/+^, *Ovch2*^−/−^, *Ovch2*^*FLAG/FLAG*^, and *Ovch2*^*Δ2FLAG/Δ2FLAG*^, and *Ovch2*^*Δ1,2/Δ1,2*^ males (*N*=2 per genotype), and performed immunoblot analyses using α-FLAG and α-OVCH2 antibodies as indicated in Figure 6. As loading control, we performed immunoblot on the same samples using α-GAPDH antibody. The precursor ADAM3 protein was detected in all the testis lysate samples at the predicted molecular weight of ~110 kDa, whereas, in the epididymis lysates, the expected cleaved ~42-kDa mature ADAM3 protein was detected in all but the *Ovch2*^−/−^ sample, which served as evidence that an abnormality in ADAM3 processing could be detected (Figure 6A). To enrich for the OVCH2-FLAG and OVCH2-Δ2FLAG proteins and to examine whether interaction with ADAM3 can be shown with mouse tissue lysates, we performed FLAG immunoprecipitation assay on testis and epididymis tissue from another set of 16-week-old *Ovch2*^*FLAG/FLAG*^ and *Ovch2*^*Δ2FLAG/Δ2FLAG*^ mice and controls (*N*=2 per genotype). OVCH2-FLAG and OVCH2-Δ2FLAG proteins could be enriched with α-FLAG mAb magnetic beads (Figure 6B). However, we did not observe specific bands for 110 kDa or 42 kDa ADAM3 proteins in the FLAG pull down samples, in either of the testes or epididymis lysates (Figure 6B). Taken together, this data suggests that while OVCH2 is required for ADAM3 processing in the mouse epididymis, its CUB domains are not, which in turn could explain our inability to recapitulate the *Ovch2* KO phenotype in the CUB1 and CUB1/2 deletant mice.

## DISCUSSION

Sperm maturation occurs in the highly regulated environment within the epididymis, where largely unknown epididymal factors govern proteolytic processing of sperm surface proteins. Up to date, growing evidence suggests that post-translational processing (maturation) of sperm surface proteins coded by germ-cell specific genes, is essential for proper sperm migration in the female reproductive tract and sperm-oocyte interaction. A known key sperm-surface protein ADAM3, also known as cyritestin, is a member of the ADAM family that belongs to the zinc protease superfamily. A complete deletion of *Adam3* in the mice leads to male sterility with impairment of sperm-UTJ transit and sperm-oocyte interaction ^50–52^. The interest in ADAM3 as a key sperm surface protein has spiked over the years due to growing evidence demonstrating that ablation of other fertility-related genes, coding for germ cell-derived, sperm-specific proteases, *Calr3, Clgn, Pdilt, Prss37, Prss55, Tmprss12*, leads to impairment of ADAM3 function, and phenocopies *Adam3* KO mice ^26,50,53^. Although genes involved in ADAM3 maturation are increasingly being identified, the underlying molecular mechanisms leading to ADAM3 processing have not yet been fully elucidated.

Previous studies proposed that sperm swim up to the oviduct through the UTJ by self-propulsion ^54^, however, many gene KO mouse lines indicate that sperm motility alone is insufficient for sperm to migrate through the UTJ, and other factors are likely involved. Several gene clusters have been discovered to be involved in regulating sperm migration through the UTJ, exhibiting elusive molecular regulatory mechanism to ensure proper sperm UTJ migration ^11,12,14,20,48,50,55–66^. Additionally, growing evidence suggests that in mice ADAM3 likely forms multiple complexes with other ADAM proteins to execute its physiological function in mouse fertility ^67^. Considering proteolytic processing of ADAM proteins in the male reproductive tract is poorly understood, elucidating the role and placement of proteases such as OVCH2 in this process is required to define the molecular and biochemical pathways that govern sperm maturation and male fertility.

Previously, we have reported that NELL2 signals through the ROS1 pathway to regulate secretion of OVCH2 into the epididymal lumen, concomitant with processing of immature ADAM3 on the sperm surface for sperm fertilizing ability ^20^. *Ovch2* deficiency in mice results in impaired migration and sperm-egg binding, as well as absence of mature ADAM3 in sperm. However, it is still unclear how OVCH2 contributes to activation of ADAM3. Previous studies have identified an array of proteins implicated in the PTMs of ADAM3, and it is likely that OVCH2, as the only known epididymal factor to date, mediates these PPIs via an unknown molecular mechanism. CUB domains, the structural components of OVCH2, have been shown to mediate PPIs in non-reproductive proteins with high substrate specificity, providing means of modulating proteolytic activity. In the present study, to clarify whether the CUB domains are involved in OVCH2 function in sperm maturation, specifically its relationship with ADAM3 maturation, we established KI/KO mice expressing OVCH2-FLAG fusion protein full-length and with CUB2 deletion and OVCH2 with both CUB domains deleted. We have found that deleting a significant portion of the OVCH2 protein (609 aa full length), one and both CUB domains with the disordered region (179 aa for CUB2 deletion and 296 aa for both CUBs) (Figure 2) did not alter the endogenous levels or tissue specificity of OVCH2 expression (Figure 3). We observed that otherwise healthy homozygous FLAG KI/CUB KO mice on average had a smaller but structurally normal and functional epididymis and exhibited subfertility early into the test mating study (Figure 4). However, upon an extended fertility assessment over the period of 6 months we did not find a significant fertility impairment, and the underlying cause of this reproductive delay in homozygous CUB1/2 KO mice remains uncertain. It is likely that this phenomenon could be attributed to a compensatory effect of another uncharacterized epididymal factor. Interestingly, we observed a significant decrease in motility and progressive motility of sperm from *Ovch2*^*Δ1,2/Δ1,2*^ males without any effect on sperm fertilizing ability (Figure 5). The mechanism by which the CUB deletion contributed to this phenomenon remains unresolved.

Although published evidence shows physiological requirement of CUB domains in various ubiquitously expressed, soluble proteins secreted into the extracellular environment of a cell, our findings revealed that the OVCH2 CUB domains are not required for OVCH2 function in male fertility. Nevertheless, in this study we illuminated the regulatory mechanism of OVCH2 in the process of sperm maturation, demonstrating that the OVCH2 trypsin-like serine protease domain is sufficient for maintaining normal fertility levels in mice. Although yet unidentified, it is possible that we will find in the future that a significant portion of men with idiopathic sperm dysfunctions phenocopies our *Ovch2* KO model in terms of sperm UTJ migration and impaired sperm-oocyte interactions. Our findings may finally lead to improvements in clinical diagnosis of male infertility and clinical management of men with mutations in *OVCH2* and its interacting proteins, paving the way to novel strategies for the development of non-hormonal male contraceptives.

## CONCLUSION

In conclusion, our studies using FLAG-tagged *Ovch2* CUB KO mice have revealed that OVCH2 CUB domains are not essential for male fertility, while the trypsin-like serine protease domain of OVCH2 is the critical component of OVCH2 required for sperm maturation in the epididymis. Our findings provide novel insights into the treatment of the infertile male and identify the vulnerable component of OVCH2 for the further development of a non-hormonal male contraceptive.

## Supplementary Material

Fig S1 **Fig. S1: PNGase F glycan cleavage of mouse OVCH2.** FLAG immunoblot with undigested (black font) and digested (red font) caput epididymis lysates from *Ovch2* WT and homozygous *Ovch2*^*FLAG*^, *Ovch2*^*Δ2FLAG*^, *Ovch2*^*Δ1,2*^ males. (A) FLAG-specific bands show a ~20 kDa drop in MWs after PNGase F glycan cleavage in FLAG KI and CUB2 KO mice. WT sample serves as a negative control for FLAG-specific antibody, while CUB1,2 KO cannot be represented due to the unintended frame-shift mutation within the FLAG sequence. (B) OVCH2-specific bands with the antigen within the C-terminally located 179 amino acids (located within the OVCH2 CUB2 domain) show ~20 kDa drop in MWs after PNGase F cleavage in WT and FLAG OVCH2. Proteins without CUB1 and CUB2 are not represented due to the epitope loss for the anti-OVCH2 antibody. Predicted MWs for WT and mutant OVCH2 are provided and were calculated without the signal peptide sequences.

Fig S3 **Fig. S3. Mice lacking OVCH2 CUB domains remain fertile despite decreased sperm kinematic parameters.** Sperm kinematics after 15-min (A) and 90-min (B) incubation in capacitation medium of *Ovch2* WT and homozygous *Ovch2*^*FLAG*^, *Ovch2*^*Δ2FLAG*^, *Ovch2*^*Δ1,2*^ sperm. VCL = curvilinear velocity; VSL = straight line velocity; VAP = average path velocity; LIN = linearity; STR = VSL/VAP (straightness); ALH = amplitude of lateral head; WOB = wobble VAP/VCL; BCF = beat cross frequency. After a 15-minute incubation the VAP, and VSL, and BCF were significantly decreased in the Ovch2 CUB2 deletants (p-value = 0.006 and 0.01, and 0.004 respectively) while after 90-minute incubation VAP, VCL and VSL remained significantly lower in the Ovch2 CUB2 deletant mice compared to their littermates (p-value = 0.001, 0.01, and 0.009, respectively), N = 5 mice/genotype, and the data are expressed as the mean ± SE, Asterisks indicate significance levels: *P < 0.05; **P < 0.01; ***P < 0.005; ****P < 0.0005.

Table S1

Table S2

Fig S2 **Fig. S2. Mice lacking OVCH2 CUB domains undergo normal spermatogenesis.** Histological analysis of testis cross sections showing seminiferous tubules in 16-week-old *Ovch2* WT and homozygous *Ovch2*^*FLAG*^, *Ovch2*^*Δ2FLAG*^, *Ovch2*^*Δ1,2*^ males. All 12 stages of spermatogenesis are represented across each experimental and control groups (n=3). PAS/hematoxylin-stained sections show normal tissue morphology and acrosome development; the acrosome is stained dark pink with periodic-acid-Shiff, whereas other cellular and tissue components are visualized through hematoxylin/eosin staining. Scale bar (60 μm) is shown for reference.

Video S1 **Vid. S1. Mice lacking both OVCH2 CUB domains exhibit decreased sperm motility.** Video capture of sperm parameters after 15-min incubation in capacitation medium of *Ovch2* WT and homozygous *Ovch2*^*FLAG*^, *Ovch2*^*Δ2FLAG*^, *Ovch2*^*Δ1,2*^ sperm. Sperm parameters are color coded and analyzed using the Hamilton Thorne CASA algorithm. The percentage of average static sperm (red) has increased in CUB1/2 KO sperm compared to that of WT sperm. N = 5 mice/genotype mice/genotype.
