# Supplementary figures and images for: CUB domains are not required for OVCH2 function in sperm maturation in the mouse epididymis
Source: Andrology. Author manuscript; Available in PMC 2024 Mar 14. (PMC10850435; doi:10.1111/andr.13508)

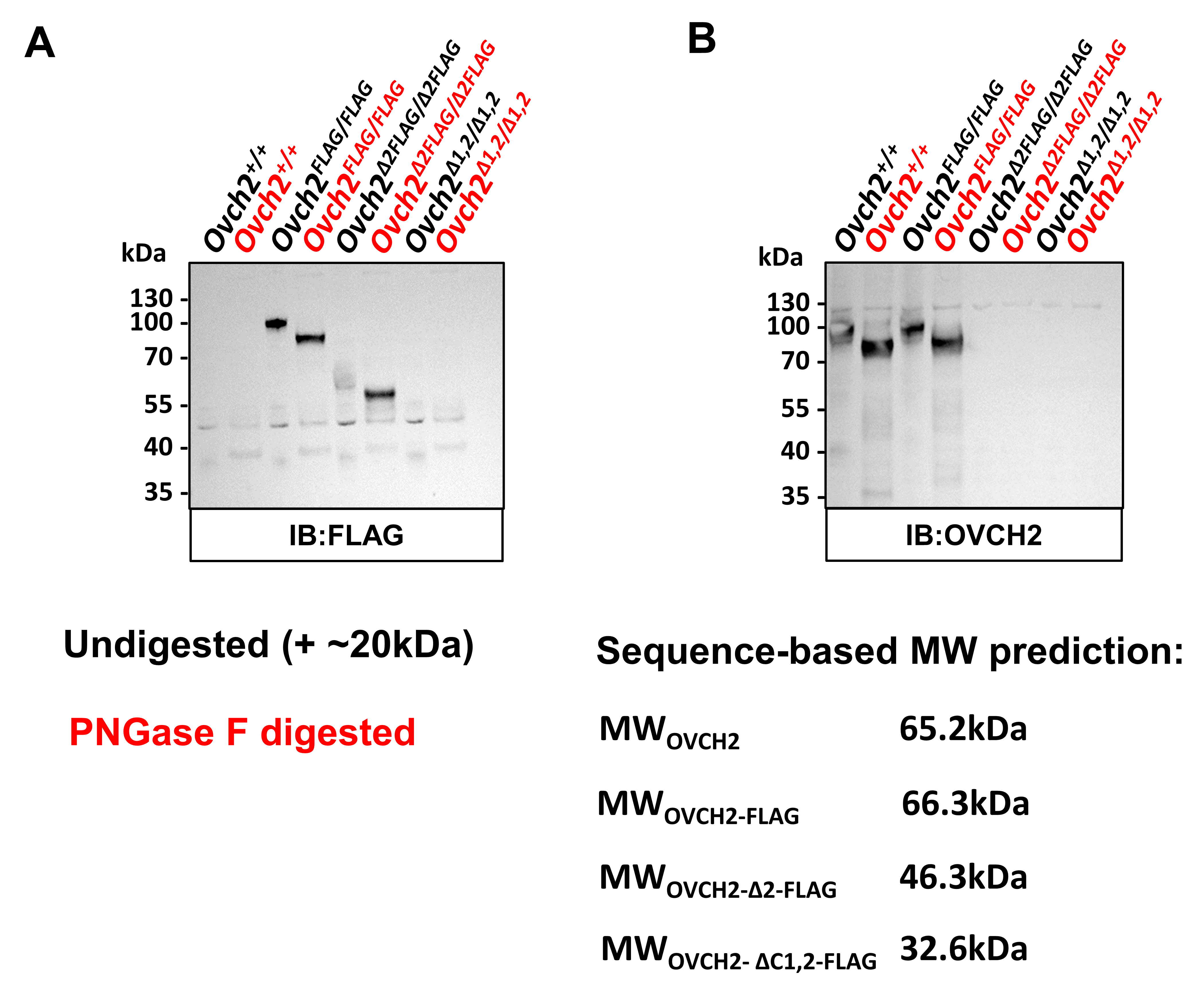

Supplement: Fig S1 — Fig. S1: PNGase F glycan cleavage of mouse OVCH2. FLAG immunoblot with undigested (black font) and digested (red font) caput epididymis lysates from Ovch2 WT and homozygous Ovch2FLAG, Ovch2Δ2FLAG, Ovch2Δ1,2 males. (A) FLAG-specific bands show a ~20 kDa drop in MWs after PNGase F glycan cleavage in FLAG KI and CUB2 KO mice. WT sample serves as a negative control for FLAG-specific antibody, while CUB1,2 KO cannot be represented due to the unintended frame-shift mutation within the FLAG sequence. (B) OVCH2-specific bands with the antigen within the C-terminally located 179 amino acids (located within the OVCH2 CUB2 domain) show ~20 kDa drop in MWs after PNGase F cleavage in WT and FLAG OVCH2. Proteins without CUB1 and CUB2 are not represented due to the epitope loss for the anti-OVCH2 antibody. Predicted MWs for WT and mutant OVCH2 are provided and were calculated without the signal peptide sequences. [file NIHMS1923368-supplement-Fig_S1.jpg]

**A**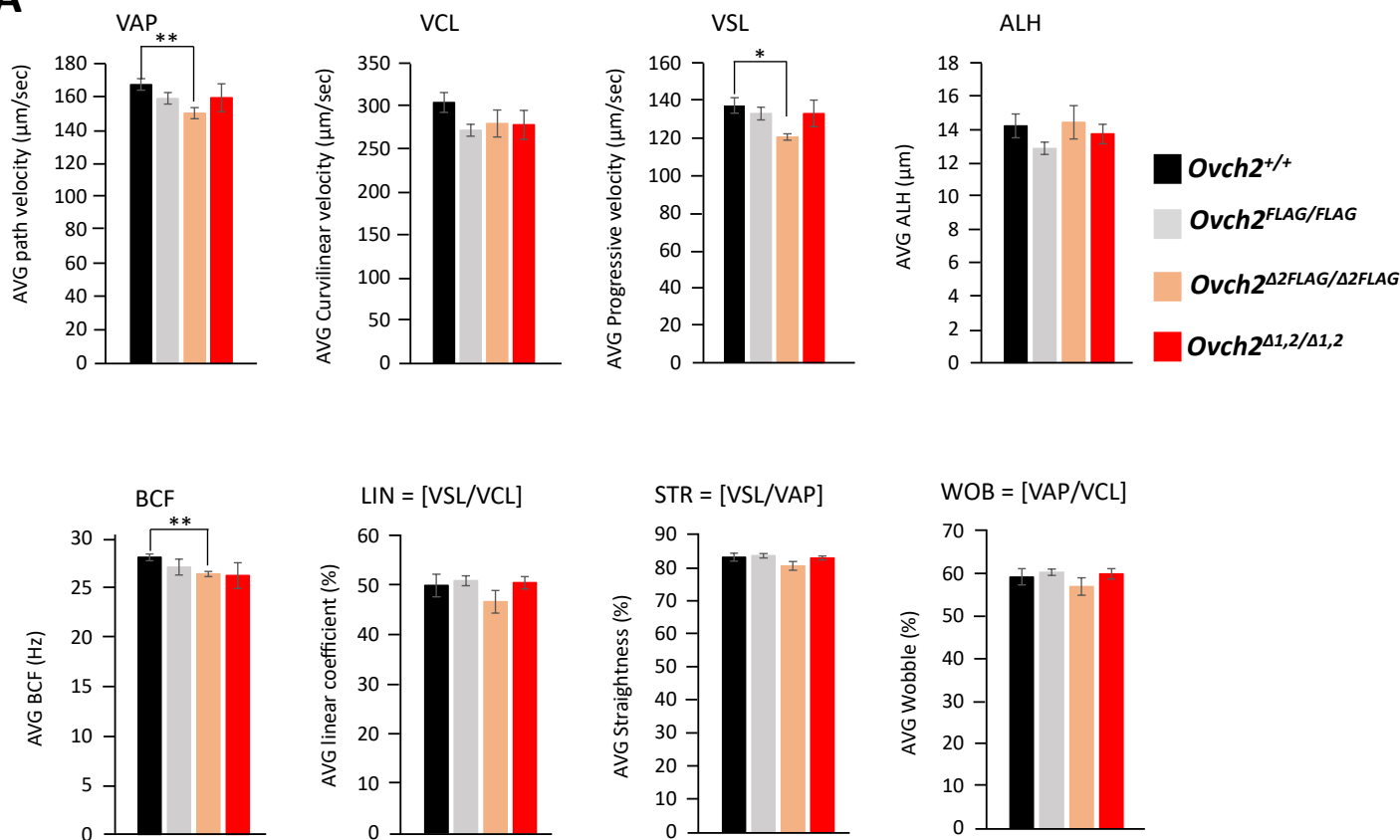**B**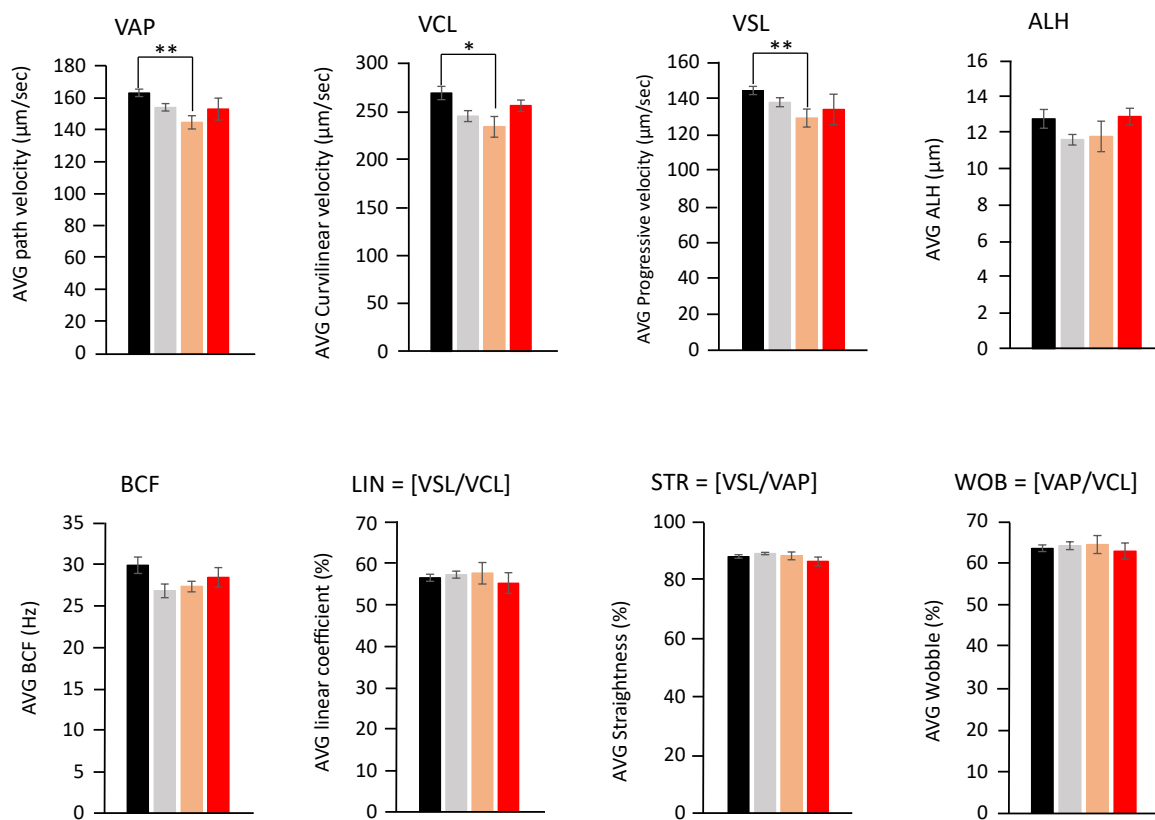

Supplement: Fig S3 — Fig. S3. Mice lacking OVCH2 CUB domains remain fertile despite decreased sperm kinematic parameters. Sperm kinematics after 15-min (A) and 90-min (B) incubation in capacitation medium of Ovch2 WT and homozygous Ovch2FLAG, Ovch2Δ2FLAG, Ovch2Δ1,2 sperm. VCL = curvilinear velocity; VSL = straight line velocity; VAP = average path velocity; LIN = linearity; STR = VSL/VAP (straightness); ALH = amplitude of lateral head; WOB = wobble VAP/VCL; BCF = beat cross frequency. After a 15-minute incubation the VAP, and VSL, and BCF were significantly decreased in the Ovch2 CUB2 deletants (p-value = 0.006 and 0.01, and 0.004 respectively) while after 90-minute incubation VAP, VCL and VSL remained significantly lower in the Ovch2 CUB2 deletant mice compared to their littermates (p-value = 0.001, 0.01, and 0.009, respectively), N = 5 mice/genotype, and the data are expressed as the mean ± SE, Asterisks indicate significance levels: *P < 0.05; **P < 0.01; ***P < 0.005; ****P < 0.0005. [file NIHMS1923368-supplement-Fig_S3.pdf]

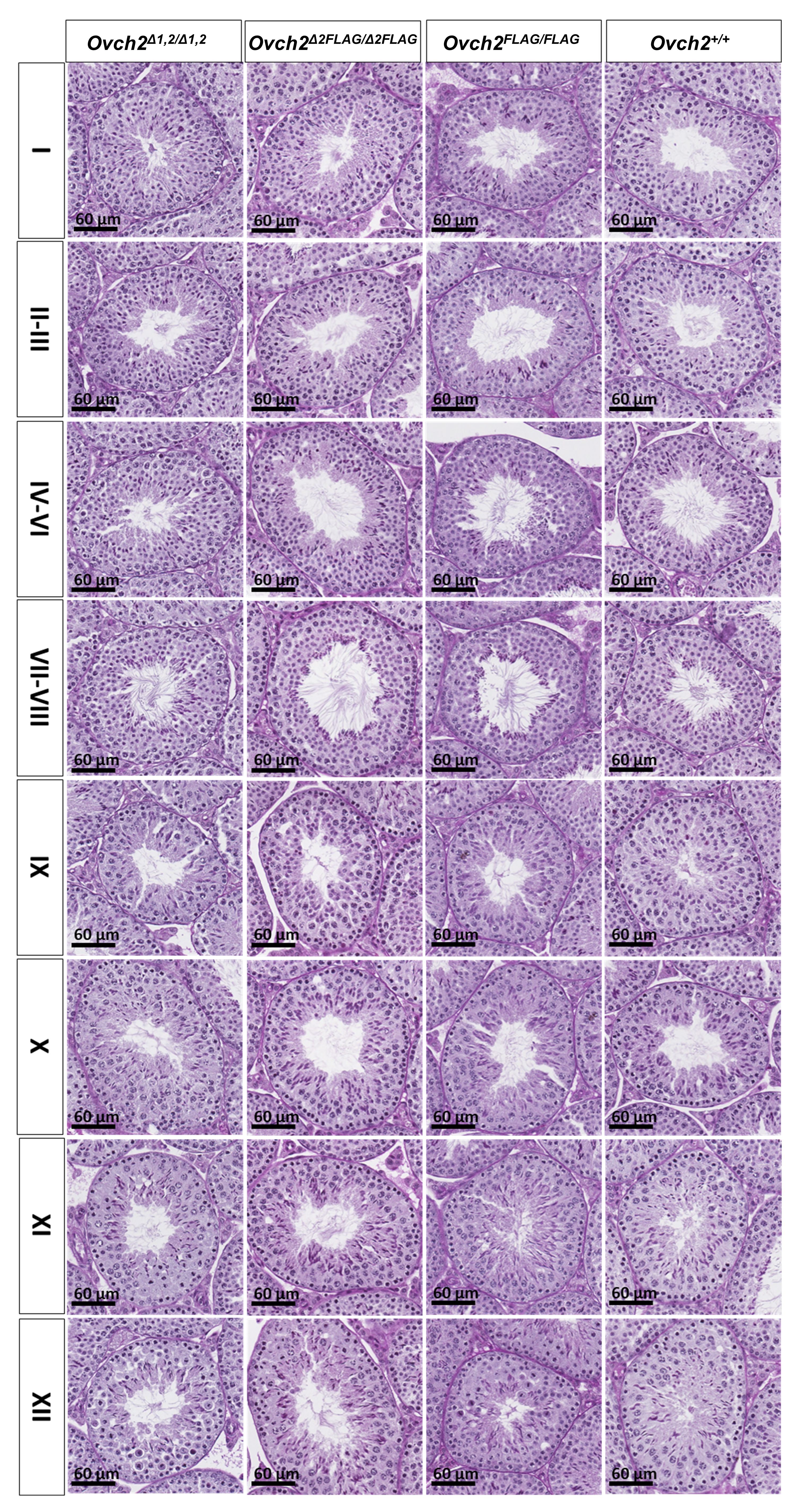

Supplement: Fig S2 — Fig. S2. Mice lacking OVCH2 CUB domains undergo normal spermatogenesis. Histological analysis of testis cross sections showing seminiferous tubules in 16-week-old Ovch2 WT and homozygous Ovch2FLAG, Ovch2Δ2FLAG, Ovch2Δ1,2 males. All 12 stages of spermatogenesis are represented across each experimental and control groups (n=3). PAS/hematoxylin-stained sections show normal tissue morphology and acrosome development; the acrosome is stained dark pink with periodic-acid-Shiff, whereas other cellular and tissue components are visualized through hematoxylin/eosin staining. Scale bar (60 μm) is shown for reference. [file NIHMS1923368-supplement-Fig_S2.jpg]
