# Supplementary material for: CUB domains are not required for OVCH2 function in sperm maturation in the mouse epididymis
Source: Andrology. Author manuscript; Available in PMC 2024 Mar 14. (PMC10850435; doi:10.1111/andr.13508)
Supplement: Table S1 [file NIHMS1923368-supplement-Table_S1.pdf]

Table S1: CRISPR/Cas9 oligo donor template with guide RNA sequences and genotyping primers for wild type and KI/KO *Ovch2* mice.

ssODN ID	ssODN sequence (5'-3')	size (nt)
OV2 FLAG KI	CCAGTGTGTCAGCAGGTTGATTCTTTCTGAGCGTTGCAATGCTTCTCATCACTTATCGTCGTCATCCTTGTAATCGGATCCATGCTTTCCACGGCTCTCCCCTTGCTTCAGAAGACCACTGTCAATAGCTGGCATCTCCCCGATGTC	147
OV2 CUB2 KO – FLAG KI	TCCTGTGTGTGTGAGTTTGTTCTGCCCTCCTTTGTTTTAGATTCCGGCGGATCCGATTACAAGGATGACGACGATAAGTGATGAGAAGCATTGCAACGCTCAGAAAGAATCAACCTGCTGACACACTGG	129
OV2 CUB1/2 KO – FLAG KI	TGCCATGCCTCATGCTGTGGCATTTCCTCTCAGCTTTATGCAGTGAGCGGATCCGATTACAAGGATGACGACGATAAGTGATGAGAAGCATTGCAACGCTCAGAAAGAATCAACCTGCTGACACACTGG	129

Guide RNA ID	Target Sequence: 5'-3' (does not include PAM)	Intended Mutation
gRNA1	GTCTTCTGAAGCAAGGGGAG	*Ovch2*^{FLAG}
gRNA2a	TTGTTTTAGATTCCGGCTGC	*Ovch2*$^{\Delta 2FLAG}$
gRNA2b	CTGAAGCAAGGGGAGAGGCG	*Ovch2*$^{\Delta 2FLAG}$
gRNA3a	GCTTTATGCAGTGAGCCTGA	*Ovch2*$^{\Delta 1,2FLAG}$
gRNA3b	CTGAAGCAAGGGGAGAGGCG	*Ovch2*$^{\Delta 1,2FLAG}$

Genotype	Forward Primer (5'-3')	Reverse Primer (5'-3')	PCR Product (bp)
Ovch2	GAGGCGTGGAAAGCATTGAG	TCAGCTCTGGACTCTGGTGA	268
Ovch2^{FLAG} (gFw1/gRe1)	CCGATTACAAGGATGACGACG	CTCTGGTGATCCCTTGGGTCT	270
Ovch2	cagCTCGGTTCTGCGACTAT	caagaacacggaatcagggc	278
Ovch2$^{\Delta 2FLAG}$ (gFw2/gRe2)	GTTCCTGATCACTGGCCCAA	GGTCTCCCTTCCTGATTGCC	448
Ovch2	AGTTCCATCCGGCTGAGGTT	AGGCAATAGTTGGGGGCTTG	575
Ovch2$^{\Delta 1,2FLAG}$ (gFw3/gRe3)	GGGTAGAACCTGGGAAGTGC	GGTCTCCCTTCCTGATTGCC	553
