# Supplementary material for: CUB domains are not required for OVCH2 function in sperm maturation in the mouse epididymis
Source: Andrology. Author manuscript; Available in PMC 2024 Mar 14. (PMC10850435; doi:10.1111/andr.13508)
Supplement: Table S2 [file NIHMS1923368-supplement-Table_S2.pdf]

Table S2: *Ovch2* cDNA-specific primers and TaqMan probes

***Ovch2* Genotype**	**Forward Primer cDNA - cFw1 (5'-3')**	**Reverse Primer cDNA - cRe1 (5'-3')**	**cDNA PCR product size (bp)**
Ovch2^{FLAG}	CCTGACTGTGGGCAGAGTTT	CTTATCGTCGTCATCCTTGTAATCGG	1767
Ovch2^{Δ2FLAG}	CCTGACTGTGGGCAGAGTTT	CTTATCGTCGTCATCCTTGTAATCGG	1230
Ovch2^{Δ1,2}	CCTGACTGTGGGCAGAGTTT	CTTATCGTCGTCATCCTTGTAATCGG	880
**Gene**	**TaqMan Assay ID**	**Catalog #**	**Mouse chromosome location**
Ovch2	Mm00556645_m1	4351372	Chr.7: 107781544 - 107801179 on Build GRCm38
Eif3l	Mm00460859_m1	4448892	Chr.15: 79075223 - 79094400 on Build GRCm38
